# Fisetin exerts neuroprotective effects *in vivo* and *in vitro* by inhibiting ferroptosis and oxidative stress after traumatic brain injury

**DOI:** 10.3389/fphar.2024.1480345

**Published:** 2024-11-20

**Authors:** Haiyi Yang, Ye Hong, Mingjie Gong, Shihong Cai, Zhongwen Yuan, Senling Feng, Qibo Chen, Xixia Liu, Zhengrong Mei

**Affiliations:** ^1^ Department of Pharmacy, Guangdong Provincial Key Laboratory of Major Obstetric Diseases, Guangdong Provincial Clinical Research Center for Obstetrics and Gynecology, The Third Affiliated Hospital of Guangzhou Medical University, Guangzhou, China; ^2^ School of Pharmaceutical Sciences, Guangzhou Medical University, Guangzhou, China; ^3^ Department of pharmacy, Guangzhou Eighth People’s Hospital of Guangzhou Medical University, Guangzhou, China; ^4^ Department of Rehabilitation, The People’s Hospital of Guangxi Zhuang Autonomous Region, Nanning, China

**Keywords:** fisetin, ferroptosis, traumatic brain injury, oxidative stress, PI3K/AKT/NRF2

## Abstract

Traumatic brain injury (TBI) is an important cause of disability and mortality, and identifying effective neuroprotective drugs and targets after TBI is an urgent public concern. Ferroptosis, an iron dependent, novel form of cell death associated with lipid peroxidation, has recently been shown to participate in secondary injury processes after TBI. Fisetin is a natural and relatively safe at general dosages flavonoid compound with neuroprotective properties. This study aimed to investigate the molecular mechanism of ferroptosis in TBI and the role of fisetin in neuroprotection by regulating ferroptosis and oxidative stress following TBI. Through *in vivo* experiments, a mouse model of repetitive mild closed head injury was established to determine that fisetin could reduce post-TBI injury and exert neuroprotective effects as determined by the Neurobehavioral Severity Scale score, brain water content, Nissl staining, hematoxylin-eosin staining, TUNEL staining and water maze experiment results. Fisetin was proven to be capable of inhibiting the changes in post-TBI ferroptosis proteins, activating the PI3K/AKT/NRF2 signaling pathway, and reducing oxidative stress, as confirmed by Western blotting. Via *in vitro* experiments, cell death models of ferroptosis were established with glutamate and erastin. As determined by MTT assay, fisetin improved the survival of cells with induced ferroptosis. The morphological alterations of ferroptotic cells were ascertained with a microscope. Fisetin similarly inhibited the changes in multiple ferroptosis-associated proteins induced by glutamate and erastin, reduced ROS and peroxidation products, and increased the level of antioxidants. In conclusion, fisetin exerts neuroprotective effects in TBI through multiple pathways, thereby alleviating tissue damage and cognitive dysfunction.

## 1 Introduction

Globally, more than 50 million people suffer from traumatic brain injury (TBI) every year, and approximately half of the world’s population has experienced at least one TBI in their lifetime (Maas et al., 2017). TBI refers to changes in brain function or other manifestations of brain pathology caused by external forces (Menon et al., 2010). Nearly half of all trauma-related deaths are caused by TBI, the leading cause of disability and death in children and young adults worldwide. The probability of epilepsy (Dulla and Pitkanen, 2021), Alzheimer’s disease (Dams-O'Connor et al., 2021), Parkinson’s disease (Gardner et al., 2018), and other neurological diseases occurring in TBI patients shows an upward trend and creates a heavy economic burden to families and societies. TBI mainly includes two basic pathophysiological processes, primary and secondary injuries. Primary injury refers to the process of immediate mechanical damage to brain tissue caused by an impact and is heterogeneous (Jassam et al., 2017). Secondary injury is a series of cascade reactions caused by the primary injury, including excitotoxicity, oxidative stress, and neuroinflammation, all of which can eventually lead to neuronal death. The interplay of a cascade of complex neurochemical and metabolic processes makes the treatment of TBI difficult (Prins et al., 2013).

Ferroptosis is an iron dependent, lipid reactive oxygen species (ROS)-accumulating form of cell death that is morphologically and biologically distinct from apoptosis, necrosis, and autophagy (Dixon et al., 2012; J; Li et al., 2020). The abnormal expression and development of various redox-active enzymes that generate or eliminate free radicals and lipid peroxidation products cause ferroptosis, resulting in an imbalance between oxidants and antioxidant products (D. Tang et al., 2021). Currently, many neurological diseases and brain injuries are associated with ferroptotic processes, such as ischemic stroke (Cui et al., 2021; C; Li et al., 2021), TBI (Rui et al., 2021; Xie B.-S. et al., 2019), and neurodegenerative diseases (Bao et al., 2021; Mahoney-Sanchez et al., 2021; Wang Z.-L. et al., 2022). Several studies have reported that the use of compounds that inhibit ferroptosis can improve the prognosis of TBI and play a neuroprotective role, such as the use of an iron uptake and transferrin receptor one inhibitor on mice with a TBI model, through which tissue damage was ameliorated by reducing iron deposition and lipid peroxidation (Cheng et al., 2022). Moreover, the injection of ferroptosis inhibitor ferrostatin-1 (Fer-1) into the ventricles of a controlled cortical impact injury mouse model reduced neuronal death and improved cognitive and motor dysfunction (Xie Y. et al., 2019). However, the mechanism of action of ferroptosis in TBI and its impact on disease progression require further investigation.

Fisetin (3,7,3′,4′-tetrahydroxyflavone) is a natural flavonoid found in a variety of vegetables, fruits, and nuts, such as strawberries, apples, persimmons, grapes, onions, and cucumbers, with the highest content in strawberries (Khan et al., 2013). Fisetin has antioxidant (Hanneken et al., 2006), anti-inflammatory (Zheng et al., 2008), and antitumor (Farooqi et al., 2021) effects and has little toxicity to normal cells (Farooqi et al., 2021; Meiyanto et al., 2012; Sundarraj et al., 2020). Previous experimental results have shown that fisetin has neuroprotective effects (Yang J. et al., 2019) and can alleviate oxidative stress after TBI (Zhang et al., 2018). However, whether fisetin exerts its therapeutic effect on TBI by inhibiting the ferroptosis pathway remains unclear.

This experiment aimed to explore the neuroprotective effect of fisetin after TBI and discover the possible molecular mechanism of ferroptosis, thereby providing new ideas and means for TBI treatment.

## 2 Materials and methods

The Third Affiliated Hospital of Guangzhou Medical University Institutional Animal Care and Use Committee approved all the experiments, which, in turn, were performed according to the guidelines of the National Institutes of Health Guide for the Care and Use of Laboratory Animals.

### 2.1 Animals

C57BL/6J mice (male, 8 weeks old) were purchased from the Guangdong Medical Laboratory Animal Center. Before the start of the experiment, the mice were adaptively reared for 7 days, and they were raised in a cycle environment with a temperature of 22°C, a humidity of 50%–60%, and light for 12 h each day and night. The mice could freely obtain food and water.

### 2.2 TBI model and drug administration

In this study, C57BL/6J mice were subjected to TBI by a modified version of the repetitive mild closed head injury as previously described (Mannix et al., 2017). Briefly, the mice were anesthetized with ether and then immobilized in the prone position on a stereotaxic apparatus. The head was placed directly under a metal hollow guide tube centered over the bregma, and a 54-g metal weight was dropped from a 28-inch-high metal hollow tube to strike the head of the mouse. Except for the mice in the sham-operated group (which received only anesthesia without injury), all mice received seven injuries over 9 days, specifically, daily blows to the head during days 1 through 5, no injury on days 6 and 7, and two more consecutive days of blows on days 8 and 9. At the end of each day’s operation, the mice were left in the fresh air of the room, and the researchers waited for them to awaken and recover.

**FIGURE 1:**
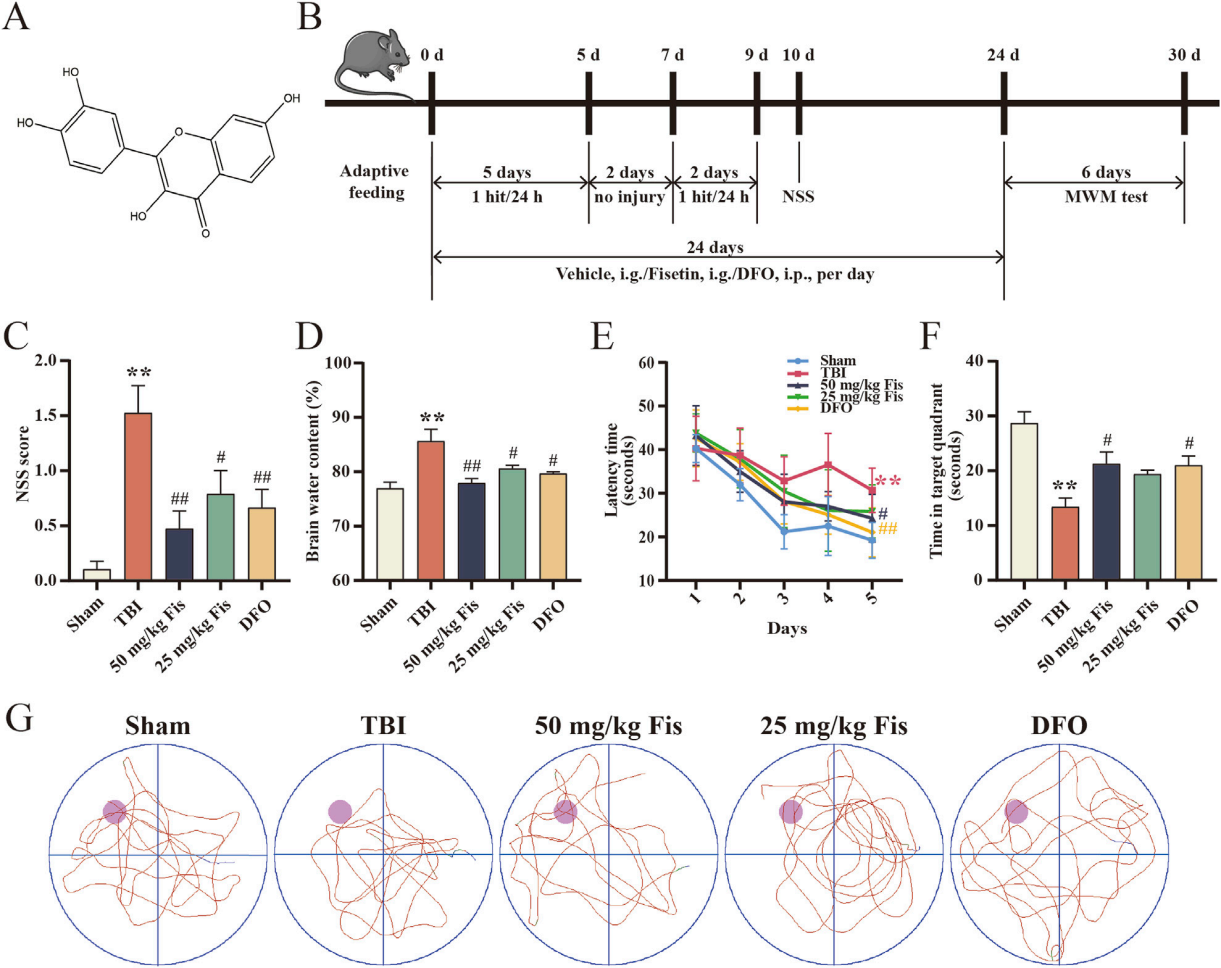
Fisetin reduces neurological damage, brain edema, and cognitive dysfunction after TBI. **(A)** Molecular structure and formula of fisetin (molecular formula: C_15_H_10_O_6_, molecular weight: 286.24). **(B)** Schematic diagram of the experimental design, including the animal model and drug delivery process. **(C)** Degree of neurological impairment represented by NSS scores (n = 15). **(D)** Brain water content of the ipsilateral hemisphere for each group (n = 3). **(E)** Time spent finding the platform in the first 5 days of the MWM tests (n = 6). **(F)** Duration of stay in the correct quadrant for 1 min in the probe test with the platform removed on day 30 of the MWM tests (n = 6). **(G)** Representative swimming trace for each group in the probe test. Data are presented as the means ± SEM. ^**^ *p* < 0.01 *versus* the sham group, ^#^ *p* < 0.05, ^##^ *p* < 0.01 *versus* the TBI group.

Before the start of the experiment, the mice were randomly divided into five groups: the sham, TBI, TBI + high dose fisetin (50 mg/kg), TBI + low dose fisetin (25 mg/kg), and TBI + DFO (100 mg/kg) groups. Deferoxamine (DFO) is an iron chelator widely used to reduce iron accumulation and deposition in tissues to inhibit cellular ferroptosis. Fisetin (Figure 1A) was dissolved in a mixed carrier of pure water + PEG400 + castor oil + absolute ethanol and administered by gavage; DFO was dissolved in physiological saline and administered by intraperitoneal injection. The treatment group mice received equal volumes of fisetin, DFO, or vehicle 30 min after daily TBI for 24 days (Figure 1B). Fisetin was purchased from Meilunbio (Dalian, China), DFO was purchased from MedChemExpress (Monmouth Junction, NJ, USA). The dose, route of administration, and experimental protocol of the drug are based on previous experimental studies and results.

### 2.3 Neurobehavioral severity scale (NSS)

To objectively quantify the degree of neurological impairment in mice on day 10, the Revised Neurobehavioral Severity Scale (NSS-R) (Yarnell et al., 2016) was used to assess the balance, motor, and reflex abilities in all mice.

### 2.4 Brain water content

On day 10, the brains of the mice in each group were removed. On a cooling matrix, the cerebellum and brainstem were rapidly dissociated and dissected, and brain tissue from the ipsilateral hemisphere was obtained. The wet weight of the ipsilateral hemisphere was measured. Then, the tissue was dried in a suitable glass dish at 80°C for 72 h and weighed again to determine its dry weight. The formula for calculating brain water content is as follows:

$$\frac{wet\;weight-dry\;weight}{wet\;weight}\times 100\%$$

### 2.5 Nissl staining

Brain tissue sections with a thickness of 6 μm were stained with Nissl staining solution, and the number and morphology of Nissl bodies in neurons in the cortical area were observed under a light microscope (Nikon, Ni-u, Tokyo, Japan).

### 2.6 Hematoxylin-eosin (H&E) staining

Brain tissue sections with a thickness of 6 μm were stained with H&E staining solution, and the number and morphology of neurons in the hippocampus were observed under a light microscope (Nikon, Ni-u, Tokyo, Japan).

### 2.7 TUNEL staining

Brain tissue sections with a thickness of 6 μm were stained with TUNEL assay reagent, the nuclei were counterstained with DAPI, and the TUNEL-positive neurons were observed under a fluorescence microscope (OLYMPUS, IX51, Tokyo, Japan).

### 2.8 Morris water maze (MWM) test

The MWM test was performed on all mice from days 25–30 to evaluate the effect of TBI on the spatial memory and learning ability of mice in each group. The MWM device consists of a constant temperature pool and a data collection and analysis system. The white circular thermostatic pool is 100 cm in diameter and 60 cm in height and is filled with water to a depth of 29 cm. The pool plane was equally divided into four quadrants, N, S, E, and W. An invisible circular escape platform was placed at the center point of one of the quadrants, 1 cm below the horizontal. In each experiment, the mice were randomly placed from the beginning of a quadrant facing the pool and given 1 min to find a hidden escape platform. The mice that found the platform within 1 min were made to stay on the platform for 15 s and then removed from the pool. The mice that failed to find the platform within 1 min were guided to the platform, made to stay there for 15 s and were then taken out of the pool. The procedure lasted for 5 days, and mice were trained 4 times a day. During day 30 of the MWM training, the hidden platform below the water level was removed, the mice were placed in a random quadrant, and the residence time of the mice in each quadrant within 1 min of being placed in the pool was observed.

### 2.9 Cell culture

Murine hippocampal HT22 cells were purchased from Procell Life Science & Technology (Wuhan, China). Cells were cultivated in DMEM containing 10% FBS, 100 U/mL penicillin, and 100 μg/mL streptomycin in a humidified incubator (5% CO_2_, 37°C).

Cells were cultured in DMEM containing 10% FBS at 37°C for 24 h and then treated with glutamate or erastin as ferroptosis inducers, followed by different concentrations of fisetin and equal volumes of ferrostatin-1 (12.5 μM), liproxstatin-1 (200 nM), and DFO (10 μM) as ferroptosis inhibitors. LY294002 (10 μM) was added independently for 1 h before treatment with glutamate or erastin. Fisetin and glutamate were purchased from Meilunbio (Dalian, China). Erastin was purchased from ApexBio (Houston, TX, USA), ferrostatin-1 and DFO were purchased from MedChemExpress (Monmouth Junction, NJ, USA), and liproxstatin-1 and LY294002 were purchased from Selleck (Shanghai, China).

### 2.10 Cell viability assay

The viability of HT22 cells was determined using MTT assay kits (Solarbio, Beijing, China). Furthermore, 100 μL of MTT solution prepared with DMEM was added to each well of a 96-well plate and cultured for 4 h. The supernatant was aspirated, 100 μL of DMSO was added to each well, and the absorbance values were measured at 490 nm after sufficient dissolution. The cell survival rate was calculated by comparing the absorbance values of the treated group with those of the drug administration group.

### 2.11 Light microscopy and transmission electron microscopy

The HT22 cells were seeded in 6-well plates and were then observed and photographed with an optical microscope (Nikon, Ti2, Tokyo, Japan) after treatment.

The HT22 cells were collected by centrifugation were fixed in 2.5% glutaraldehyde and then in 1% osmium tetroxide. After dehydration and infiltration, the samples were cut into ultrathin sections with a thickness of approximately 100 nm using a Lecia UC7 ultramicrotome and double acetic acid. The samples were then stained with uranyl and lead citrate and observed and photographed under a transmission electron microscope (FEI, Tecnai G2 Spirit, Czechia).

### 2.12 Western blot analysis

Mouse brain tissue or HT22 cells were lysed with RIPA lysis buffer (strong) containing a protease inhibitor cocktail and a phosphatase inhibitor cocktail (CWBIO, Beijing, China) for 20 min on ice and centrifuged at 14,000 rpm for 10 min at 4°C. The supernatant was subsequently collected. Protein concentrations were determined with the Pierce BCA Protein Assay Kit (Thermo, USA). Total protein extracts (20 μg) were separated by SDS-PAGE, transferred to PVDF membranes (Millipore, Burlington, MA, USA), and blocked with 5% skim milk for 90 min. The membrane was incubated with primary antibodies against GPX4 (glutathione peroxidase 4, 1:2000, ab125066, Abcam), SLC7A11 (xCT, the functional component of system Xc-, 1:1000, 12,691, CST), SLC3A2 (4F2hc, the functional component of system Xc-, 1:1000, 47,213, CST), FTH1 (ferritin heavy chain, 1:2000, 4393, CST), DMT1 (divalent metal transporter 1, 1:1000, 15,083, CST), NCOA4 (nuclear receptor coactivator 4, 1:1000, 66,849, CST), PRDX1 (peroxiredoxin 1, 1:2000, ab109498, Abcam), AKT (1:2000, 4691, CST), p-AKT (1:2000, 4060, CST), PI3K (1:2000, 41,339, SAB), p-PI3k (1:2000, 12,057, SAB), NRF2 (1:2000, ab31163, Abcam), β-Tubulin (1:3000, 01,270, CWBIO) overnight at 4°C. After three washes in Tris-buffered saline with Tween, the membranes were incubated with diluted horseradish peroxidase-conjugated anti-rabbit or anti-mouse secondary antibodies (1:3000, CWBIO) for 60 min at room temperature. The protein bands were detected by enhanced chemiluminescence (Millipore, Burlington, MA, USA). Digital images of the protein bands were recorded by Chemidoc MP (Bio-Rad, Hercules, CA, USA), and their gray values were analyzed by ImageJ software.

### 2.13 Iron assay kit

According to the iron assay manual (Abcam, Cambridge, UK), the brain tissue is homogenized in the buffer and mixed with the reagents. The mixture is then incubated, after which the iron probe is added and incubated in the dark at 37°C for 60 min. The iron content is determined using colorimetric methods.

### 2.14 SOD activity and MDA content

According to the instructions, the SOD and MDA levels of HT22 cells were detected by a Total Superoxide Dismutase Assay Kit with WST-8 and a Lipid Peroxidation MDA Assay kit (Beyotime, Shanghai, China), respectively.

### 2.15 8-OHdG concentrations

The ELISA kit (CUSABIO, Wuhan, China) detects the concentration of 8-OHdG in tissues. The microtiter plate provided in this kit has been pre-coated with an 8-OHdG antibody. Samples are added to the appropriate microtiter plate wells along with a Horseradish Peroxidase conjugated antibody preparation specific for 8-OHdG. A competitive inhibition reaction occurs between the pre-coated 8-OHdG and the 8-OHdG present in the samples. The level of 8-OHdG is calculated based on absorbance.

### 2.16 Glutathione measurement

The HT22 cells were collected and added to RIPA buffer lysate containing a protease inhibitor cocktail for 20 min. Centrifugation was performed at 14,000 rpm for 10 min at 4°C, and then the concentration was measured with the Pierce BCA Protein Assay Kit. Subsequently, 100 μL of the supernatant, 100 μL of NEM (50 mM), and 500 μL of prechilled methanol (50 ng/mL Phe-d5) were mixed and placed in a refrigerator at −20°C for 2 h. The solution was centrifuged at 15,000 rpm for 15 min at 4°C. Then, 500 μL of supernatant was collected and redissolved in 100 µL of aqueous methanol (20 vol%) after drying at 30°C using a nitrogen stream. After centrifugation at 15,000 rpm for 15 min at 4°C, 80 µL of the supernatant was collected. The GSH content was detected by LC‒MS/MS, and the analytical method for LC-MS/MS was based on previous experiments.

### 2.17 ROS production

According to the Reactive Oxygen Species Assay Kit (Biosharp, Hefei, China), HT22 cells were incubated with serum-free medium containing DCFH-DA for 20 min. After washing with PBS, the cells were digested and collected. A Flow Cytometry (Thermo, Waltham, MA, USA) BL -1 channel was then used for analysis.

### 2.18 Statistical analysis

The results were expressed and analyzed as the means ± standard error of the mean (SEM). All data were analyzed using GraphPad Prism 9.0 (La Jolla, CA, USA) and SPSS 17.0 (SPSS, Inc., Chicago, IL) software. Statistical comparisons were made using one-way analysis of variance. A value of *p* less than 0.05 was considered to show a significant difference between the groups.

## 3 Results

### 3.1 Fisetin attenuates neuron injury after TBI

To determine whether fisetin treatment could alleviate neurological dysfunction after TBI, we assessed the changes by the NSS. On day 1 after the last injury, the NSS score of the TBI group was significantly higher than that of the sham group, and the scores of the TBI + fisetin and TBI + DFO groups were decreased compared to those of the TBI group, and no significant difference was found between the 50 mg/kg and 25 mg/kg fisetin treatment groups (Figure 1C). Furthermore, brain water content was significantly increased in the TBI group compared with the sham group. Edema of the ipsilateral brain was also alleviated after fisetin or DFO treatment. The effect of 50 mg/kg fisetin in reducing cerebral edema was also more significant (Figure 1D). Therefore, both fisetin and DFO attenuated the level of neuronal damage after TBI.

### 3.2 Fisetin attenuates cognitive impairment after TBI

To assess the level of cognitive function in mice following TBI, we used the MWM test to determine whether fisetin could mitigate TBI-induced decline in learning and memory. The TBI group took more time to reach the hidden platform than the sham group on days 25–29. In contrast, the TBI +50 mg/kg fisetin group showed a shorter latency period than the TBI group from day 28, but the TBI +25 mg/kg fisetin group showed a significant difference only on day 28 (Figure 1E). In addition, the probe experiment on day 30 revealed that TBI mice stayed in the target quadrant corresponding to the platform for significantly less time than the sham group, but the 50 mg/kg fisetin-administered group increased the residence time of TBI mice in the target quadrant (Figure 1F). In both observations, DFO exhibited a rescue effect on cognitive impairment after TBI. The representative swim trace for each group is indicated in Figure 1G.

### 3.3 Fisetin promotes neuronal survival after TBI

**FIGURE 2:**
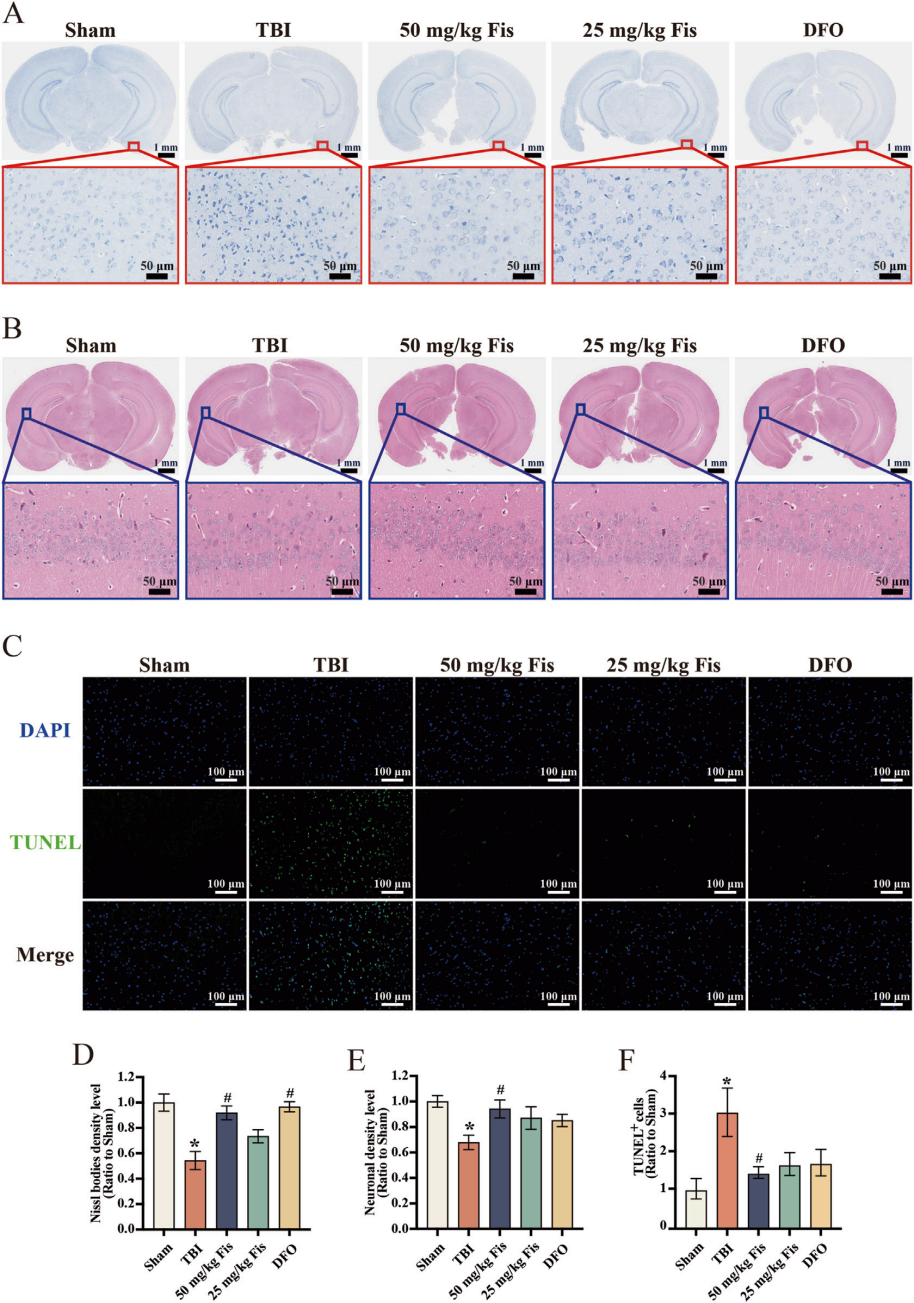
Fisetin mitigates histopathological changes and promotes neuronal survival after TBI. **(A)** Representative images of cortical tissue via Nissl staining. Scale bars = 1 mm and 50 μm. **(B)** Representative images of the hippocampus of the CA1 regions via H&E staining. Scale bars = 1 mm and 50 μm. **(C)** Representative images of cortical tissue via TUNEL fluorescence staining. Scale bars = 100 μm. **(D)** Quantitative analyses of Nissl bodies density (n = 3). **(E)** Quantitative analyses of neuronal density level (n = 3). **(F)** Quantitative analyses of TUNEL^+^ cells (n = 3). Data are presented as the means ± SEM. ^*^ *p* < 0.05 *versus* the sham group, ^#^ *p* < 0.05 *versus* the TBI group.

To further determine the effect of fisetin on morphological changes in brain tissue after TBI, we used Nissl staining and H&E staining to examine changes in neuronal morphology and number. The injured neurons in Nissl staining shrank, exhibiting irregular cell bodies and hyperchromatic nuclei (H. Wang et al., 2020) in the vicinity of the damaged cortical area. Relative to the sham group, the TBI group exhibited a significantly higher number of damaged neurons, and the arrangement was scattered. The 50 mg/kg fisetin-treated groups mitigated these changes (Figure 2A). Using H&E staining to examine the hippocampal CA1 region, we found that neurons in the Sham group were neatly arranged, structurally intact, and clearly stained. However, in the TBI model group, the neurons were sparsely arranged and disordered, and the nerve fibers were reduced. In contrast, the 50 mg/kg fisetin-administered groups exhibited reduced TBI neuronal loss and increased neuronal survival (Figure 2B). Ferroptosis can lead to double-strand breaks in DNA, resulting in positive TUNEL staining (Linkermann et al., 2014; Wang et al., 2022). We used TUNEL staining to assess the degree of cell death in the sections and observed that the TBI group had significantly more TUNEL^+^ cells than the Sham group. In contrast, the 50 mg/kg fisetin group showed a reduction in positive cell numbers compared to the TBI group. (Figure 2C). The statistical histogram presents the results of the quantitative analysis of Nissl bodies, neuronal density, and TUNEL^+^ cells (Figures 2D–F).

### 3.4 Fisetin inhibits TBI-induced ferroptosis

**FIGURE 3:**
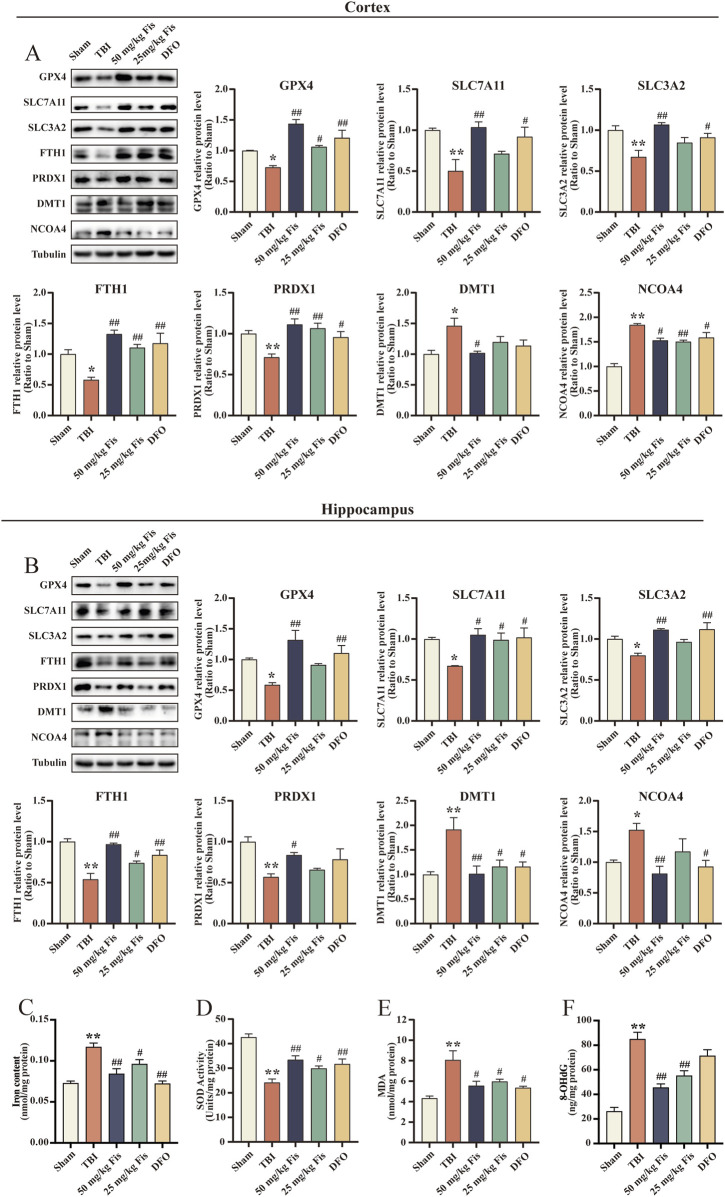
Fisetin inhibits ferroptosis and oxidative stress after TBI. **(A, B)** Expression levels of GPX4, SLC7A11, SLC3A2, FTH1, PRDX1, DMT1, and NCOA4 proteins in cortical and hippocampal tissues as detected by Western blot. The levels were normalized to β-tubulin (n = 3). **(C)** Iron content in the cortical tissue (n = 3) **(D)** SOD activity levels in cortical tissues (n = 3). **(E)** MDA levels in the cortical tissue (n = 3). **(F)** 8-OHdG content in the cortical tissue (n = 3). Data are presented as the means ± SEM. ^*^ *p* < 0.05, ^**^ *p* < 0.01 *versus* the sham group, ^#^ *p* < 0.05, ^##^ *p* < 0.01 *versus* the TBI group.

To verify whether the therapeutic effect of fisetin on TBI is related to ferroptosis, we first detected the expression of the ferroptosis-related proteins GPX4, SLC7A11, SLC3A2, FTH1, DMT1, NCOA4, and PRDX1 in the cortex and hippocampus by Western blotting. The expression of GPX4, SLC7A11, and SLC3A2 was significantly decreased in the TBI model group compared to the sham group, but the fisetin-administered groups mitigated this change. We also detected a significant decrease in the expression levels of FTH1 and PRDX1 in the TBI group and an upward trend in the levels of DMT1 and NCOA4. The iron content assay shows that the brain tissue iron levels in the TBI model group are significantly increased. Meanwhile, the fisetin-administered group suppressed these changes. Interestingly, the TBI + DFO group exhibited similar effects to the fisetin groups (Figures 3A–C). These results suggest that TBI is accompanied by ferroptosis and that fisetin can inhibit TBI-induced ferroptosis.

### 3.5 Fisetin attenuates TBI-induced oxidative damage

Given the close association between oxidative stress and ferroptosis, we next examined the levels of MDA and SOD in cortical tissues. As expected, the activity of the antioxidant enzyme SOD in the cortical tissue of the mice in the TBI model group was significantly reduced, and the level of MDA and 8-OHdG—an indicator of DNA oxidative damage—were markedly increased, indicating an exacerbation of lipid peroxidation. In contrast, the oxidative damage to the cortex was alleviated by fisetin intervention with an increase in SOD activity and a decrease in MDA levels and 8-OHdG content. This outcome is consistent with the trend of changes in the DFO treatment group (Figures 3D–F).

### 3.6 Fisetin enhances survival in the glutamate- and erastin-induced HT22 cell model of ferroptosis

**FIGURE 4:**
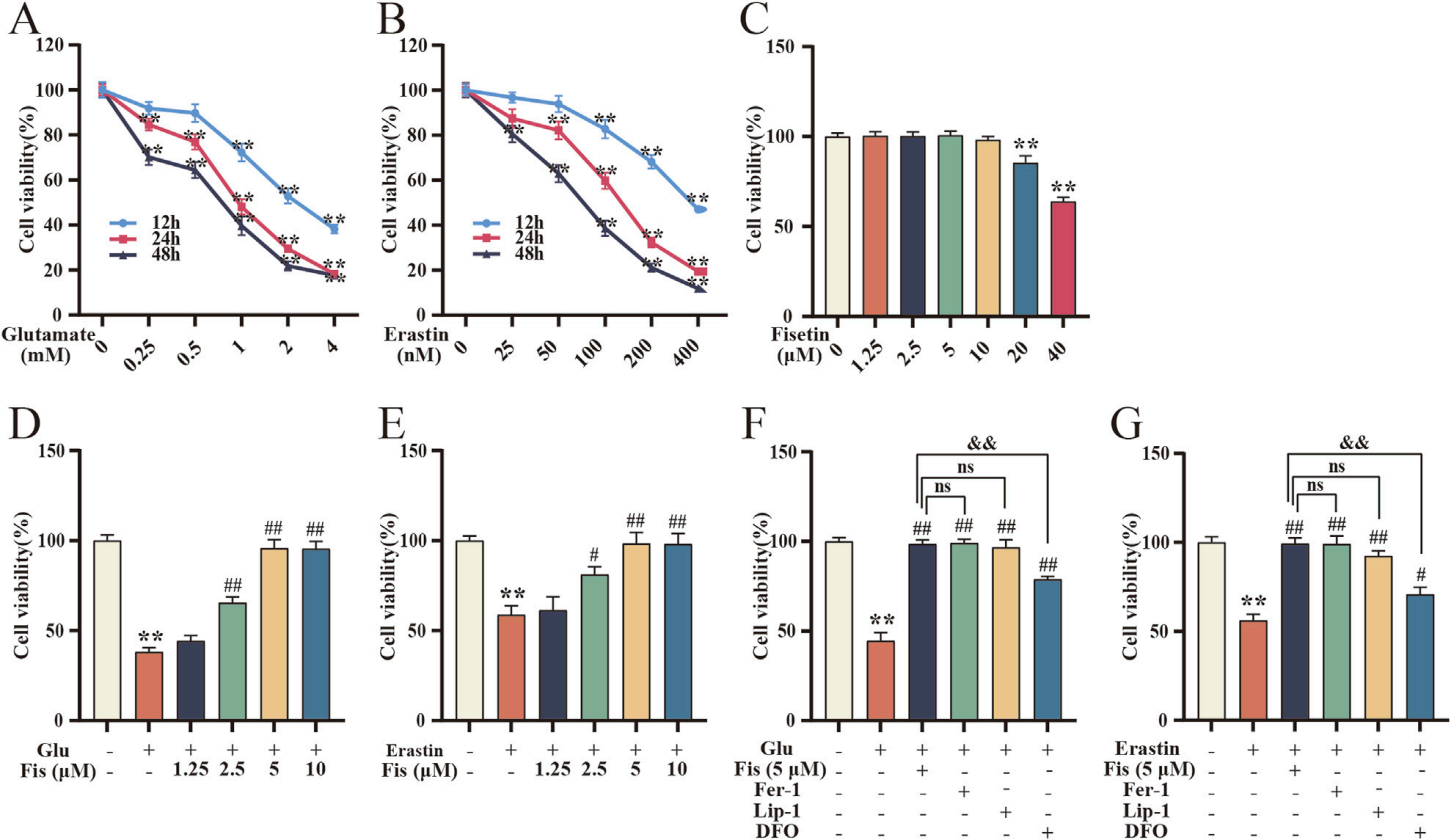
Fisetin enhances survival in the glutamate- and erastin-induced HT22 cell model of ferroptosis. **(A, B)** Changes in HT22 cell viability with the concentration and duration of action of glutamate and erastin by MTT assay. Data are presented as the means ± SEM. ^**^ *p* < 0.01 *versus* the control group (n = 3). **(C)** Effect of different concentrations of fisetin on HT22 cell viability at 24 h by MTT assay. Data are presented as the means ± SEM. ^**^ *p* < 0.01 *versus* the control group (n = 3). **(D, E)** Rescue of cell viability of HT22 cells induced by glutamate and erastin at 24 h with different concentrations of fisetin by MTT assay (n = 3). Data are presented as the means ± SEM. ^**^ *p* < 0.01 *versus* the control group, ^#^ *p* < 0.05, ^##^ *p* < 0.01 *versus* the glutamate or erastin group. **(F, G)** Effect of fisetin on glutamate- and erastin-induced HT22 cell viability at 24 h compared with Fer-1, Lip-1, and DFO by MTT assay (n = 3). Data are presented as the means ± SEM. ^**^ *p* < 0.01 *versus* the control group, ^#^ *p* < 0.05, ^##^ *p* < 0.01 *versus* the glutamate or erastin group, ^&&^ *p* < 0.01 *versus* the glutamate +5 μM fisetin or erastin +5 μM fisetin group.

To further explore the inhibitory effect and action pathway of fisetin on ferroptosis, we chose the ferroptosis inducers erastin and glutamate to establish a ferroptosis model in HT22 cells. First, HT22 cells were exposed to a series of concentration gradients of erastin or glutamate. Both erastin and glutamate decreased cell viability in time- and dose-dependent manners, respectively. Treatment of the HT22 cells with 0.1 μM erastin for 24 h reduced cell viability to 53.8% ± 3.4%, and the counterpart with 1 mM glutamate reduced cell survival to 49.5% ± 1.9% (Figures 4A, B). Thus, 0.1 μM erastin and 1 mM glutamate treatment for 24 h were used for subsequent experiments. Next, we assessed the effect of fisetin on the viability of HT22 cells by MTT assay. Fisetin below 10 μM did not show significant cytotoxicity at 24 h (Figure 4C). Different concentrations of fisetin were also coincubated with erastin and glutamate. Compared with the model group without fisetin treatment, fisetin improved cell viability, and the effect was optimally significant when the concentration was 5 μM (Figures 4D, E). In addition, cotreatment of HT22 cells with ferrostatin-1, liproxstatin-1, or DFO with erastin or glutamate for 24 h resulted in a significant increase in cell viability. Notably, 5 μM fisetin mitigated cell death better than DFO (Figures 4F, G). Therefore, the use of erastin and glutamate can establish a cell ferroptosis model and reduce cell viability, and fisetin can increase cell viability and inhibit cell death.

### 3.7 Fisetin reduces morphological changes in the glutamate- and erastin-induced ferroptosis model in HT22 cells

**FIGURE 5:**
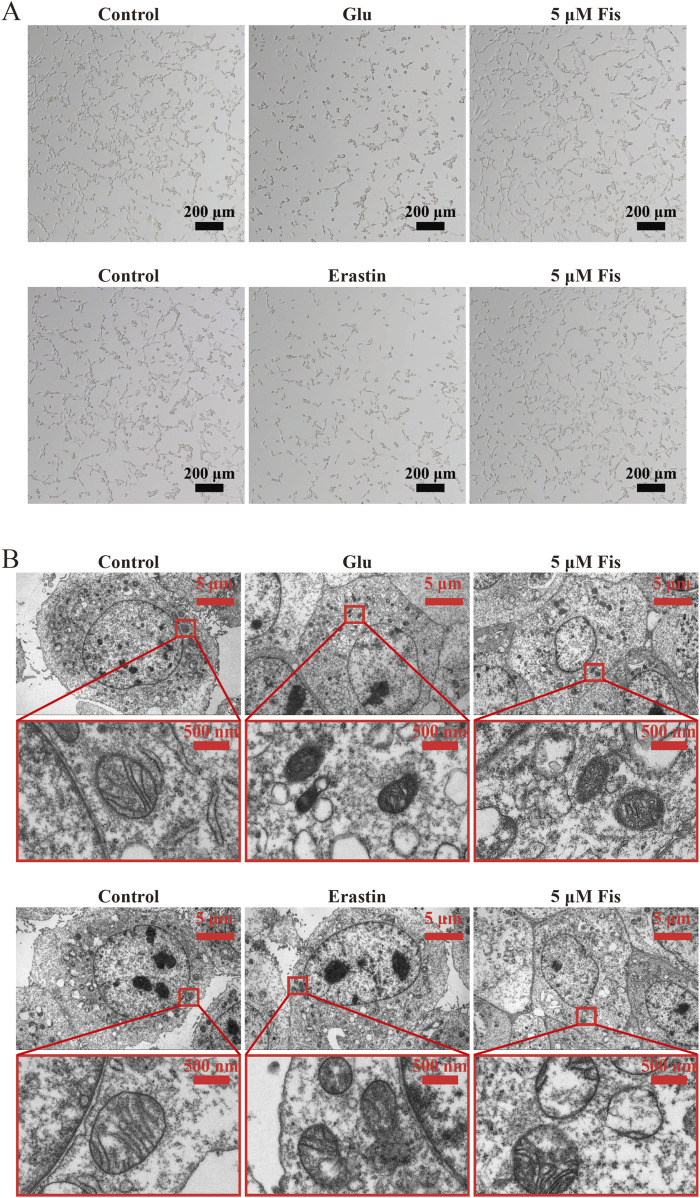
Fisetin reduces morphological changes in glutamate- and erastin-induced ferroptosis in HT22 cells. **(A)** Morphological changes in HT22 cells induced by the administration of fisetin on glutamate and erastin as observed under a light microscope. Scale bar = 200 μm. **(B)** Morphological changes in mitochondria in HT22 cells induced by fisetin administration on glutamate and erastin as observed under a transmission electron microscope. Scale bars = 5 μm and 500 nm.

Cell morphology was observed under a light microscope. Compared with those of the control group, the cells treated with erastin or glutamate alone had reduced neurite formation, and some cells became rounded and lost their neuron-like morphology. However, in the 5 μM fisetin-treated group, the junctions between cells were tight, and the number of cells in suspension decreased (Figure 5A). Morphological changes in mitochondria are also regarded as important features of ferroptosis. The mitochondria of the HT22 cells observed under a transmission electron microscope revealed that the model group treated with erastin or glutamate showed a decrease in mitochondrial volume, a decrease in the number of ridges, and an increase in membrane density, but the addition of 5 μM fisetin inhibited this change (Figure 5B). These results further demonstrate that fisetin inhibits ferroptosis and reduces morphological changes induced by erastin and glutamate.

### 3.8 Fisetin reduces glutamate- and erastin-induced changes in ferroptosis-related proteins in HT22 cells

**FIGURE 6:**
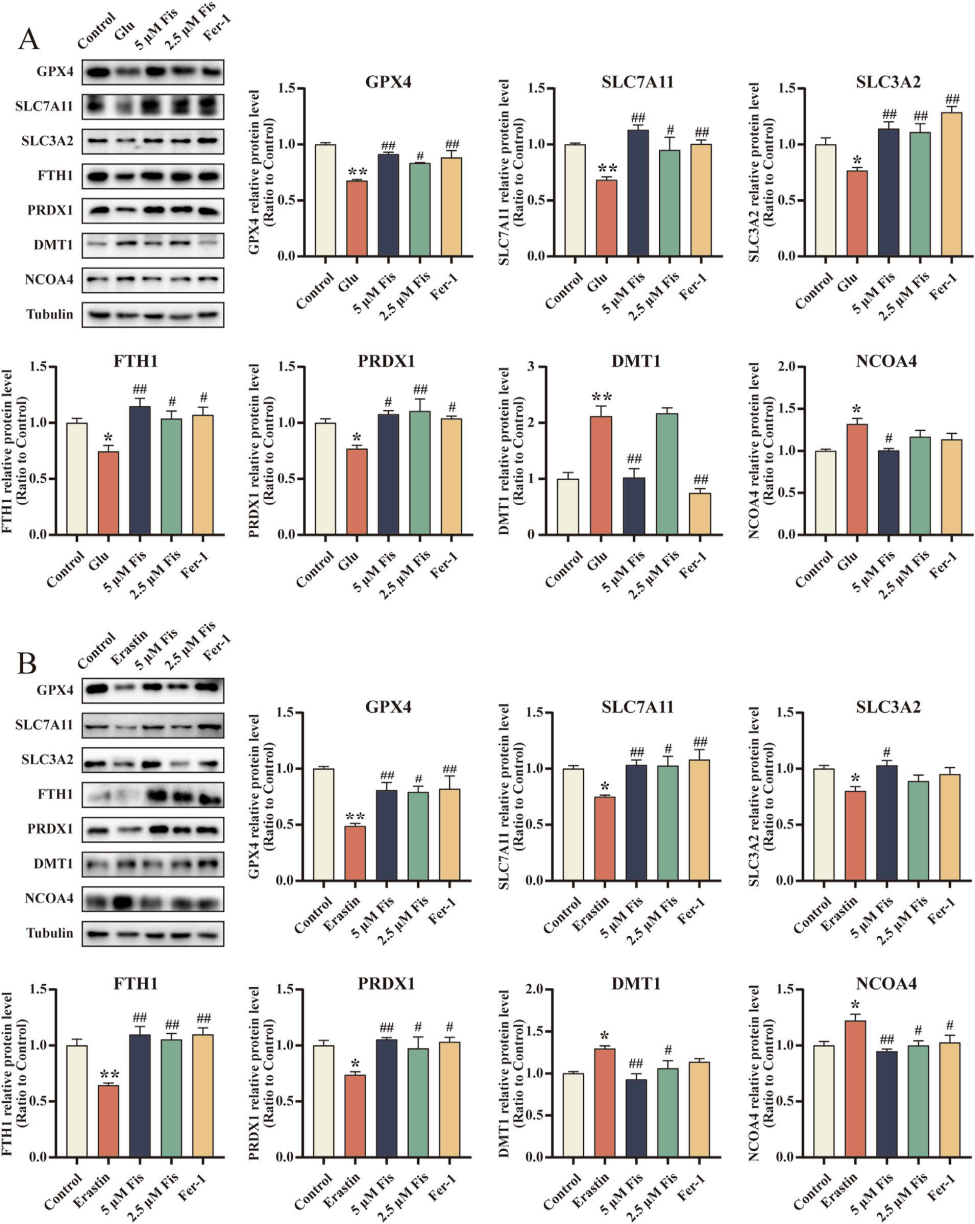
Fisetin reduces glutamate- and erastin-induced changes in ferroptosis-related proteins in HT22 cells. **(A, B)** Expression levels of GPX4, SLC7A11, SLC3A2, FTH1, PRDX1, DMT1, and NCOA4 in HT22 cells induced with glutamate and erastin and in response to fisetin at 24 h by Western blot. The levels were normalized to β-tubulin (n = 3). Data are presented as the means ± SEM. ^*^ *p* < 0.05, ^**^ *p* < 0.01 *versus* the control group, ^#^ *p* < 0.05, ^##^ *p* < 0.01 *versus* the glutamate or erastin group.

Likewise, we examined the effect of fisetin on the protein levels of erastin- and glutamate-induced HT22 cells. Similar to the outcome of the *in vivo* experiments, the levels of GPX4, SLC7A11, SLC3A2, FTH1, and PRDX1 in cells treated with erastin or glutamate alone were significantly decreased, while the expression levels of DMT1 and NCOA4 were significantly increased (Figures 6A, B). The administration of fisetin reversed these changes, an outcome comparable to that after the administration of Fer-1 treatment.

### 3.9 Fisetin inhibits glutamate- and erastin-induced oxidative stress in HT22 cells and attenuates lipid peroxidation levels

**FIGURE 7:**
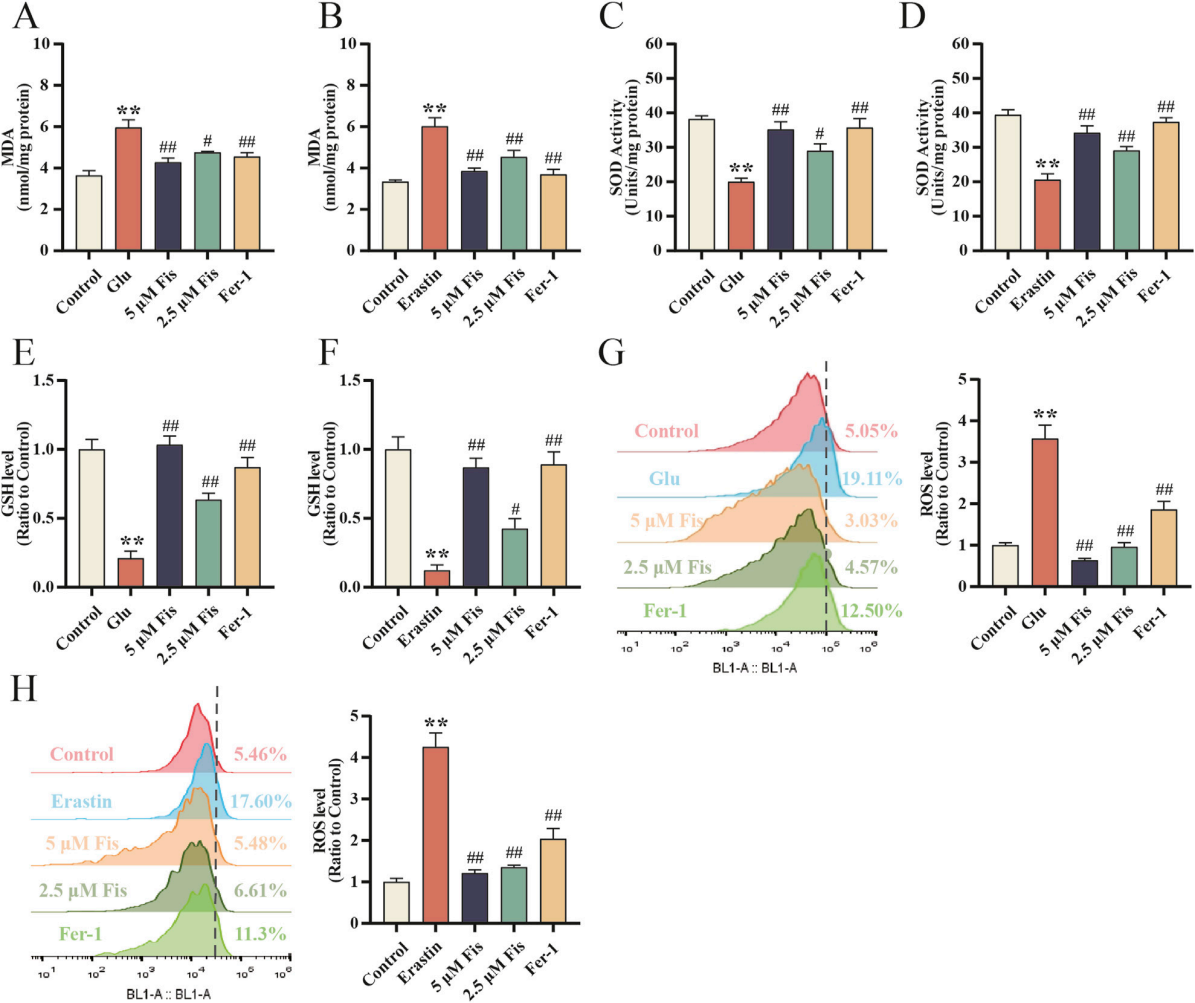
Fisetin inhibits erastin- and glutamate-induced oxidative stress in HT22 cells and attenuates lipid peroxidation levels. **(A–H)** MDA, SOD, GSH, and ROS levels in HT22 cells induced with glutamate and erastin and in response to fisetin at 24 h (n = 3). Data are presented as the means ± SEM. ^**^ *p* < 0.01 *versus* the control group, ^#^ *p* < 0.05, ^##^ *p* < 0.01 *versus* the glutamate or erastin group.

Compared with the control group, the erastin and glutamate groups exhibited significantly increased ROS and MDA levels. Compared with the model group, the erastin + fisetin, erastin + Fer-1, glutamate + fisetin and glutamate + Fer-1 groups showed decreased levels of MDA (Figures 7A, B) and ROS (Figures 7G, H), thereby reducing the extent of cellular lipid peroxidation. In addition, the levels of SOD (Figures 7C, D) and GSH (Figures 7E, F) in the erastin and glutamate groups were significantly decreased, but fisetin and Fer-1 increased the levels of SOD and GSH, respectively, indicating that fisetin enhanced the antioxidant enzyme activity of ferroptotic cells and reduced oxidative stress to play a neuroprotective role.

### 3.10 Fisetin inhibits ferroptosis via the PI3K/AKT/NRF2 pathway

**FIGURE 8:**
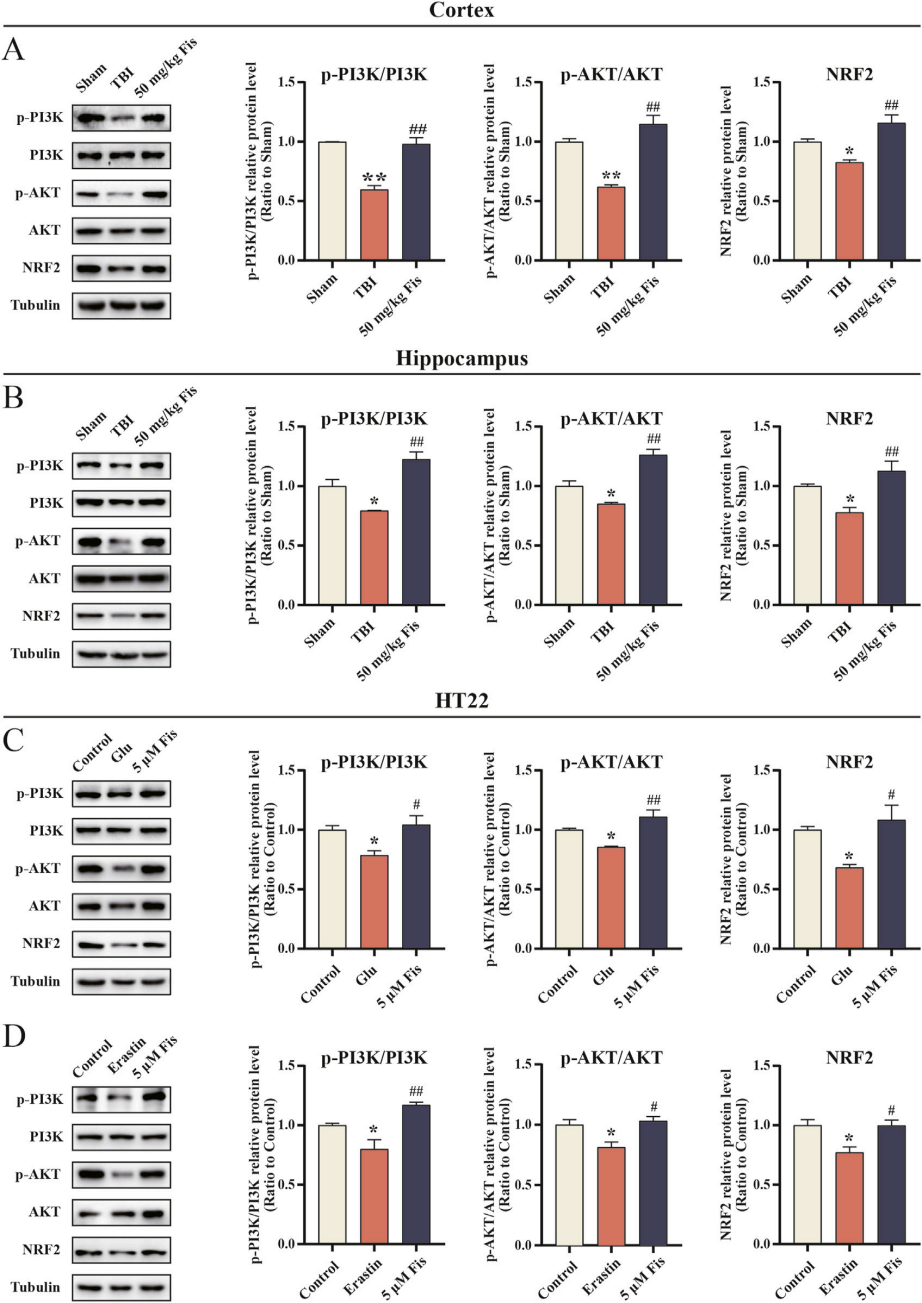
Fisetin activates the PI3K/AKT/NRF2 pathway in both post-TBI cortex and hippocampal tissue and in HT22 cells induced by glutamate and erastin. **(A, B)** Expression levels of p-PI3K/PI3K, p-AKT/p-AKT, and NRF2 proteins in cortical and hippocampal tissues were detected by Western blot. The levels were normalized to β-tubulin (n = 3). ^*^ *p* < 0.05, ^**^ *p* < 0.01 *versus* the sham group, ^##^ *p* < 0.01 *versus* the TBI group. Data are presented as the means ± SEM. **(C, D)** Expression levels of p-PI3K/PI3K, p-AKT/AKT, and NRF2 protein levels in HT22 cells induced with glutamate and erastin and in response to fisetin at 24 h as determined by Western blot. The levels were normalized to β-tubulin (n = 3). ^*^ *p* < 0.05 *versus* the control group, ^#^ *p* < 0.05, ^##^ *p* < 0.01 *versus* the glutamate or erastin group. Data are presented as the means ± SEM.

To investigate whether the inhibitory effect of fisetin on ferroptosis involves the PI3K/AKT/NRF2 pathway, we examined protein expression in both *in vivo* and *in vitro* models. Western blot analysis showed that in the *in vivo* experiments, the levels of p-PI3K/PI3K, p-AKT/AKT, and NRF2 were significantly decreased in the cortical and hippocampal tissues of the TBI model group but were increased in the fisetin-treated group compared with the model group (Figures 8A, B). Similarly, in the *in vitro* experiments, administration of erastin or glutamate alone downregulated the levels of p-PI3K/PI3K, p-AKT/AKT, and NRF2 in HT22 cells compared to the control group, but fisetin intervention reversed these changes (Figures 8C, D).

**FIGURE 9:**
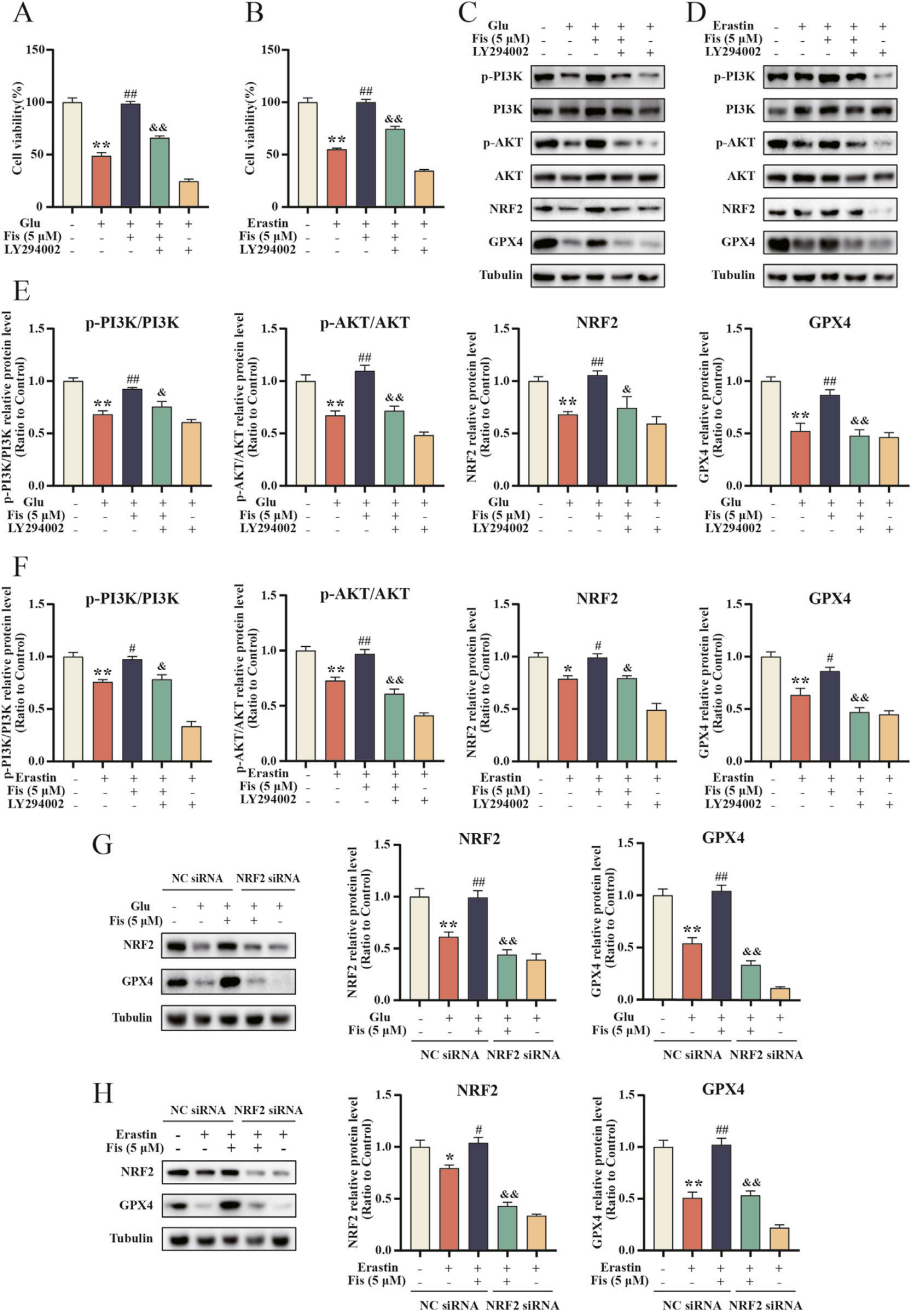
Fisetin attenuates glutamate- and erastin-induced ferroptosis by the PI3K/AKT/NRF2 pathway in HT22 cells. **(A, B)** LY294002 reduces the protective effect of fisetin on the glutamate- and erastin-induced cell viability of HT22 cells at 24 h by MTT assay (n = 3). Data are presented as the means ± SEM. ^**^ *p* < 0.01 *versus* the control group, ^##^ *p* < 0.01 *versus* the glutamate or erastin group, ^&&^ *p* < 0.01 *versus* the glutamate +5 μM fisetin or erastin +5 μM fisetin group. **(C–F)** Effects on fisetin activation of the glutamate- and erastin-induced PI3K/AKT/NRF2 signaling pathway in HT22 cells after using LY294002 as determined by Western blot (n = 3). Data are presented as the means ± SEM. ^*^ *p* < 0.05, ^**^ *p* < 0.01 *versus* the control group, ^#^ *p* < 0.05, ^##^ *p* < 0.01 *versus* the glutamate or erastin group, ^&^ *p* < 0.05, ^&&^ *p* < 0.01 *versus* the glutamate +5 μM fisetin or erastin +5 μM fisetin group. **(G, H)** HT22 cells were transfected with NRF2 siRNA, and the effect of fisetin on the expression levels of NRF2 and GPX4 protein in glutamate- and erastin-induced cells was determined by Western blot (n = 3). Data are presented as the means ± SEM. ^*^ *p* < 0.05, ^**^ *p* < 0.01 *versus* the control group, ^#^ *p* < 0.05, ^##^ *p* < 0.01 *versus* the glutamate or erastin group, ^&&^ *p* < 0.01 *versus* the glutamate +5 μM fisetin or erastin +5 μM fisetin group.

To determine the crucial effect of PI3K/AKT/NRF2 on the mechanism of ferroptosis inhibition by fisetin, HT22 cells were treated with the PI3K/AKT inhibitor LY294002. Cell viability was first assayed by MTT, and consistent with previous results, cell viability was significantly increased in the fisetin-administered group compared to the glutamate and erastin groups. However, the survival rate was significantly lower in the LY294002 pretreatment group than in the fisetin-administered group, thereby reversing the protective effect of fisetin (Figures 9A, B).

Next, we examined the effect of PI3K/AKT inhibition on NRF2 and GPX4 protein changes. The fisetin group exhibited increased expression of the NRF2 and GPX4 proteins compared to the glutamate and erastin groups. However, pretreatment with LY294002 significantly decreased NRF2 and GPX4 protein expression and increased cellular ferroptosis and oxidative stress levels (Figures 9C–F). Thus, fisetin regulates the PI3K/AKT/NRF2 signaling pathway, increases the expression levels of the NRF2 and GPX4 proteins, inhibits ferroptosis and oxidative stress in cells, and protects neural cells from damage.

To further evaluate the role of NRF2 in fisetin protecting HT22 cells from ferroptosis induced by glutamate and erastin, NRF2 siRNA transfection was used to silence NRF2 in HT22 cells. The results showed that the silencing of NRF2 largely reversed the increased expression of GPX4 protein induced by fisetin, counteracting the inhibitory effect of ferroptosis. In conclusion, the inhibitory effect of fisetin on ferroptosis is partly dependent on the PI3K/AKT/NRF2 signaling pathway.

## 4 Discussion

According to reports, fisetin is a natural nontoxic flavonol molecule widely present in various diets. Fisetin has attracted much attention because of its efficacy in models of stroke (Wang L. et al., 2019), memory disorders (S. Ahmad et al., 2021), depression (Y. Wang et al., 2017), and several neurodegenerative diseases, including Alzheimer’s and Parkinson’s (Khan et al., 2013; Nabavi et al., 2016; Ravula et al., 2021). Fisetin can scavenge free radicals, increase intracellular GSH content, and has a high antioxidant capacity (Khan et al., 2013). One of the main contributors to secondary injury following TBI is oxidative stress. Given their high levels of polyunsaturated lipids, high oxygen consumption, and poor antioxidant capacity, neurons are extremely vulnerable to oxidative stress. (Liu et al., 2017; Rego and Oliveira, 2003). Targeted reduction of oxidative stress is critical for neuronal survival and recovery of neurological function after TBI. However, few studies on the effect and mechanism of fisetin on TBI are available, and this scarcity aroused our attention.

**FIGURE 10:**
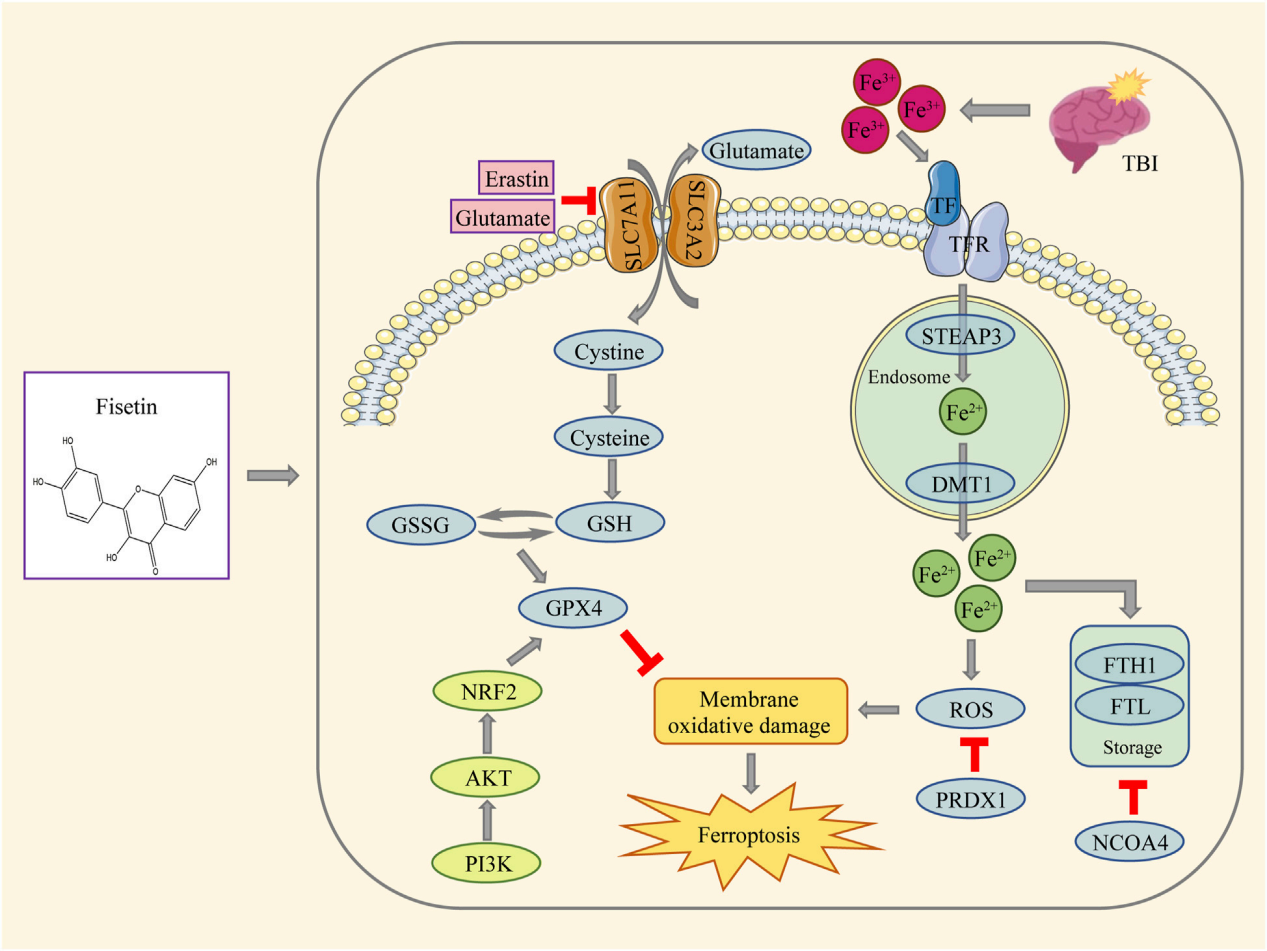
Mechanisms of fisetin on ferroptosis and oxidative stress in glutamate- and erastin-stimulated HT22 cells and rats after TBI, which may involve the PI3K/AKT/NRF2 pathway. Fisetin mitigates lipid peroxidation in ferroptosis by increasing the levels of SLC7A11, SLC3A2, GPX4, and reduced glutathione. At the same time, fisetin activates PI3K/AKT/NRF2 pathway after injury, which may be closely related to the neuroprotective effect of fisetin in relieving oxidative stress. In addition, fisetin can also increase the expression level of FTH1 and PRDX1 proteins, and reduce the expression of DMT1 and NCOA4, thereby reducing ROS level, inhibiting lipid peroxidation, alleviating ferroptosis, and protecting neurons. Fisetin inhibits ferroptosis and oxidative stress *in vivo* and *in vitro* through a variety of ways.

In the mouse repetitive mild closed head injury model and the HT22 cell model induced by erastin and glutamate, fisetin exerted neuroprotective effects by regulating ferroptosis through the PI3K/AKT/NRF2 pathway. Generally, mechanical stretch injury and oxygen-glucose deprivation are common *in vitro* cell models for TBI (Hou et al., 2024; Keating and Cullen, 2021). However, these models struggle to replicate all the pathological changes and cascading responses that occur during TBI (Salvador et al., 2015), and they do not meet the requirements for investigating the pharmacological effects of the compounds on ferroptosis in brain tissue during TBI. Therefore, this study utilized ferroptosis inducers to establish the cell model. Our results proved that 1) fisetin reduces brain water content, attenuates neuron damage and cognitive dysfunction after TBI, and plays a neuroprotective role. 2) Ferroptosis occurs after TBI, and the administration of fisetin can inhibit changes in the ferroptosis marker GPX4 and other oxidative stress indicators, such as MDA and SOD, remove excess oxygen free radicals, and mitigate the occurrence of lipid peroxidation. 3) Fisetin also has an inhibitory effect on ferroptosis inducers in in vitro cell experiments. 4) The inhibitory effect of fisetin on ferroptosis is related to the PI3K/AKT/NRF2 pathway (Figure 10).

A plasma membrane-associated lipid kinase called PI3K is made up of the regulatory subunits p85 and p55 as well as the catalytic subunit p110. (Donahue et al., 2012; Yang W. et al., 2019). After phosphorylation, PI3K can change the protein structure of AKT and activate it. All AKT isoforms are highly expressed in the nervous system and can protect nerves from injury (Rai et al., 2019). PI3K/AKT is engaged in a number of processes, including cell proliferation, differentiation, apoptosis, oxidative stress, and inflammation. It plays a significant role in mediating neuronal survival under a variety of situations. (Choudhury et al., 2017; Xie B.-S. et al., 2019). Nerve growth factor (NGF), insulin-like growth factor 1 (IGF-1), brain-derived neurotrophic factor (BDNF), and other trophic factors all play a role through PI3K/AKT pathway (Rai et al., 2019). At present, the reduction of post-TBI injury by regulating the PI3K/AKT pathway has been explored in studies as follows: histone deacetylases maintain microglial function and protect white matter after TBI through the PTEN/PI3K/AKT signaling pathway (G. Wang et al., 2015), and the flavonoid wogonin exerts antioxidative and antiapoptotic effects and reverses neuronal damage after TBI through the PI3K/AKT pathway (Feng et al., 2022). Consistent with previous research, this work confirms that PI3K/AKT is closely related to the TBI injury mechanism. In TBI, the administration of fisetin can simultaneously upregulate the activated phosphorylation levels of PI3K and AKT in the cortex and hippocampus, activate downstream NRF2 translocation to the nucleus, inhibit the occurrence of ferroptosis, and play antioxidation and anti-injury roles. Moreover, fisetin can also reverse the occurrence of ferroptosis and improve cell survival by activating the PI3K/AKT signaling pathway in glutamate- and erastin-induced cell injury models. Fisetin-induced p-AKT phosphorylation markedly attenuated NRF2 accumulation, decreased inhibition of changes in ferroptosis-associated proteins, and reduced cell viability after administration of the PI3K inhibitor LY294002.

As an important antioxidant regulator, NRF2 plays a significant protective role in TBI (H. Wang et al., 2020; Wang J. et al., 2019). When oxidative stress occurs in the secondary injury of TBI, NRF2 dissociates from KEAP-1 in the cytoplasm, translocates to the nucleus, accumulates in the nucleus, and plays an antioxidant role by activating downstream response elements. Existing experimental results confirmed that fisetin can reduce oxidative stress after TBI by activating the NRF2-ARE pathway (Zhang et al., 2018). However, the trend of NRF2 expression after brain injury is controversial. For example, Gao et al. reported elevated NRF2 content at 24 h after injury in mice using a cortical impactor (Gao F. et al., 2021), and Wang et al. reported elevated NRF2 content at 72 h in a TBI mouse model induced by weight drop (H. Wang et al., 2020). Conversely, Bhowmick et al. showed a significant decrease in NRF2 content at 24 h after a TBI model induced by the fluid percussion injury method in mice (Bhowmick et al., 2019), and Ahmad et al. reported that NRF2 expression was inhibited after TBI injury caused by using a sharp-edged scalpel blade (R. Ahmad et al., 2022). A reason for this difference in expression may arise from disparities in the manner and extent of damage caused to brain tissue and the time of measurement, resulting in variations in NRF2 activation levels.

Iron dyshomeostasis and ferroptosis are highly involved in the secondary injury process of TBI (S. Tang et al., 2020). Inhibition of ferroptosis can reduce TBI tissue damage and improve long-term prognosis (Xie Y. et al., 2019). Reduction of iron dyshomeostasis and lipid peroxidation presents a viable treatment for TBI. The only known reductase capable of reducing lipid peroxidation in biological membranes is GPX4. Additionally, GPX4 can convert harmful lipid peroxides into harmless lipo alcohols (Bersuker et al., 2019; Ingold et al., 2018; Yang et al., 2014). and is widely regarded as a key regulator of ferroptosis. GPX4-deficient mice exhibit hippocampal neurodegeneration and cognitive impairment (Hambright et al., 2017). In this study, the expression level of GPX4 protein decreased significantly after 3 days of TBI, a result that is in line with the findings of earlier studies (Wang D. et al., 2022). Treatment with fisetin was able to reverse this change. Similarly, upstream of the GPX4 protein, a cystine/glutamate antiporter, can mediate the transport of extracellular cystine into the cell and convert cystine into cysteine to participate in the synthesis of reduced glutathione to maintain proper cell functioning (Bridges et al., 2012; Chen et al., 2021) and play an important role in the regulation of ferroptosis. Furthermore, the absence of SLC7A11 or SLC3A2 reduces GSH production and inhibits GPX4 activity to induce ferroptosis. Other ferroptosis-related proteins, such as FTH1, DMT1, and NCOA4, also affect the development of ferroptosis. Prior research confirmed that the occurrence of ferroptosis in the Alzheimer’s disease mouse model was accompanied by a significant decrease in SLC7A11, SLC3A2, and FTH1 and an increase in the protein expression of DMT1 and NCOA4 (Gao Y. et al., 2021). This remarkable finding led us to test the expression of these ferroptosis-related proteins and the effect of fisetin administration on these factors. Western blot results showed that the expression of SLC7A11, SLC3A2, and FTH1 decreased and the expression of DMT1 and NCOA4 increased in TBI; crucially, fisetin inhibited these changes. These outcomes suggest that ferroptosis plays an important role in the secondary injury of TBI and that fisetin can play a neuroprotective role by inhibiting ferroptosis. In addition, PRDX1 has been shown to inhibit lipid peroxidation (Lovatt et al., 2020), and the reduction of PRDX1 results in the formation of cellular ROS and the induction of ferroptosis. (Luo et al., 2022). We sought to ascertain whether the PRDX1 level in the brain tissue would change after TBI and verified the following results: the PRDX1 level in the TBI model group decreased significantly, and the PRDX1 protein expression level rose after fisetin treatment. Through *in vitro* cell experiments, we also verified the changes in the expression trends of all the above proteins. Interestingly, GPX4, SLC7A11, FTH1, and PRDX1 are all considered downstream targets of NRF2 (Anandhan et al., 2020; Dodson et al., 2019; Lovatt et al., 2020). However, whether these changes in ferroptosis proteins are directly related to the loss of the NRF2 protein remains unclear.

## 5 Conclusion

In summary, this study confirms that fisetin can alleviate tissue damage and cognitive dysfunction after TBI. Fisetin can inhibit ferroptosis *in vivo* and *in vitro*, and this neuroprotective mechanism may be related to the PI3K/AKT/NRF2 pathway. These findings provide a new direction for the treatment strategy and target of TBI.

## Data Availability

The original contributions presented in the study are included in the article/Supplementary Material, further inquiries can be directed to the corresponding authors.
