# Supplementary material for: Fisetin exerts neuroprotective effects in vivo and in vitro by inhibiting ferroptosis and oxidative stress after traumatic brain injury
Source: Front Pharmacol. 2024 Nov 20;15:1480345. doi: 10.3389/fphar.2024.1480345 (PMC11615404; doi:10.3389/fphar.2024.1480345)

Supplementary Material

# Supplementary Figures and Tables

# Transfection efficiency

**
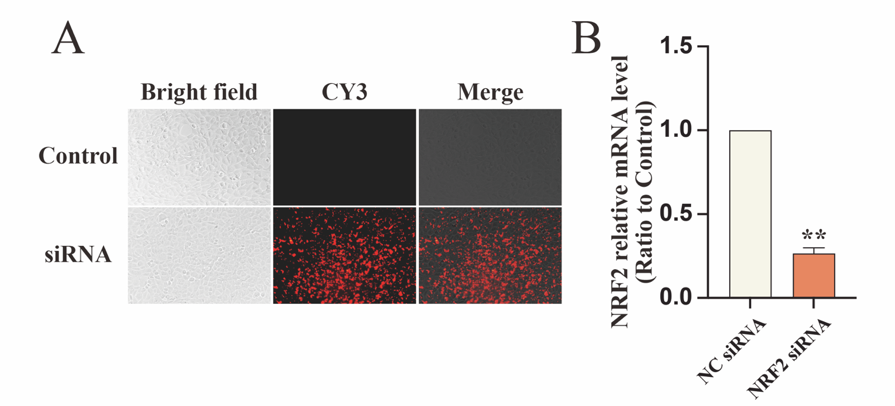
**

(A) Fluorescence map of siRNA transfected HT22 cells. (B) Interference efficiency of siRNA NRF2 transfection into HT22 cells. **P<0.01 versus the Control group.

# WB corresponding loading band


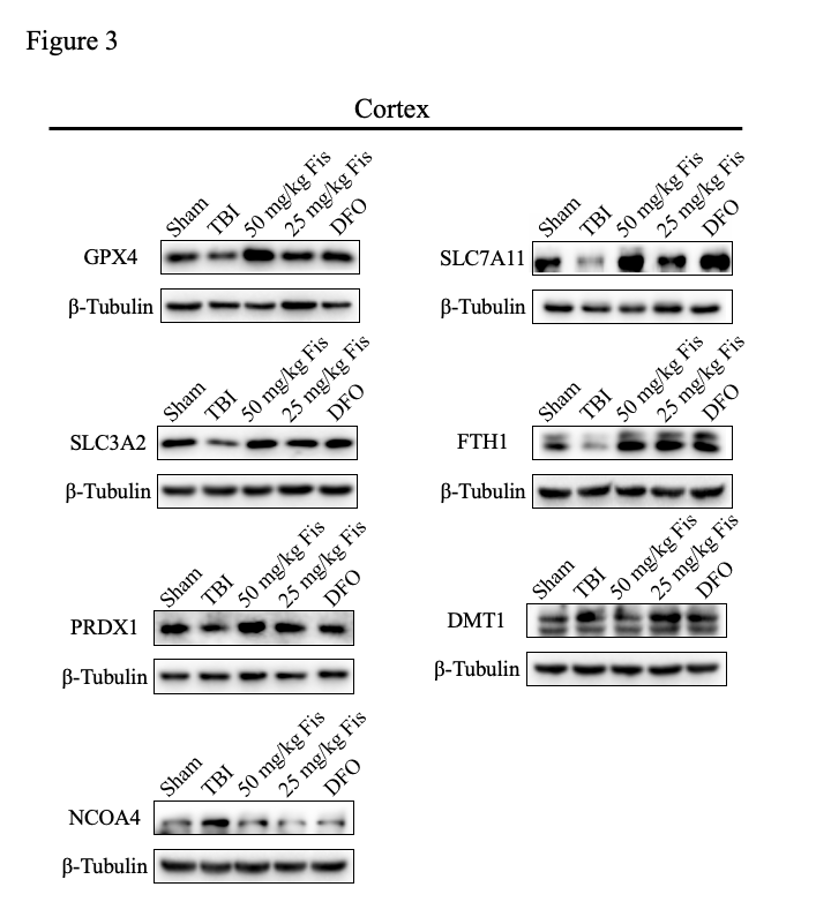


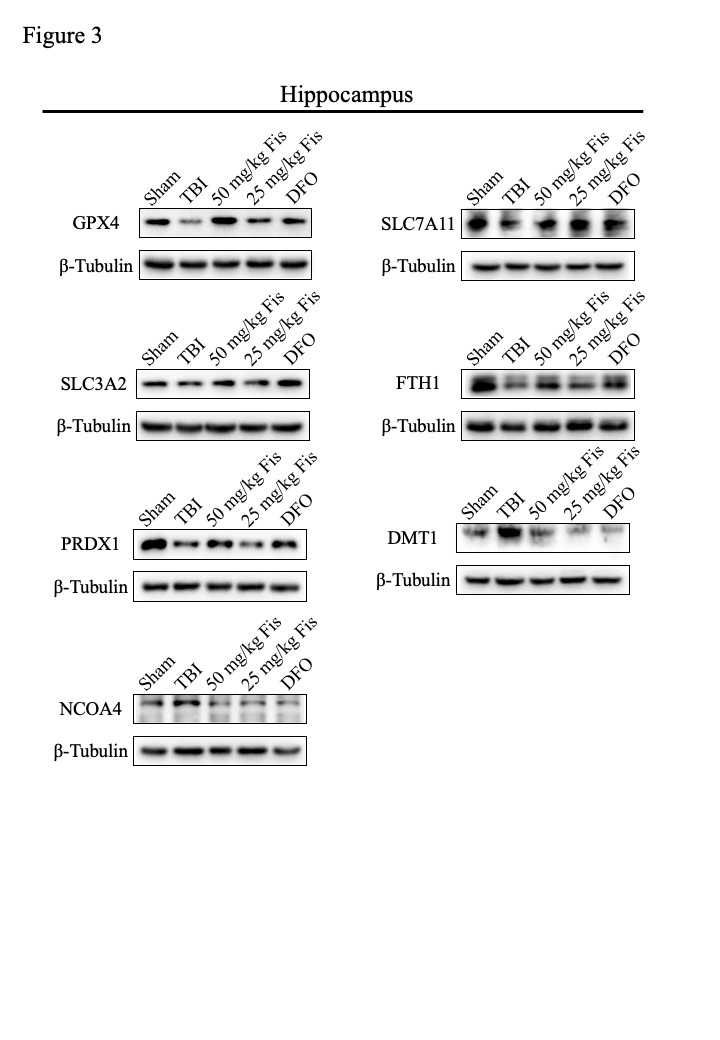


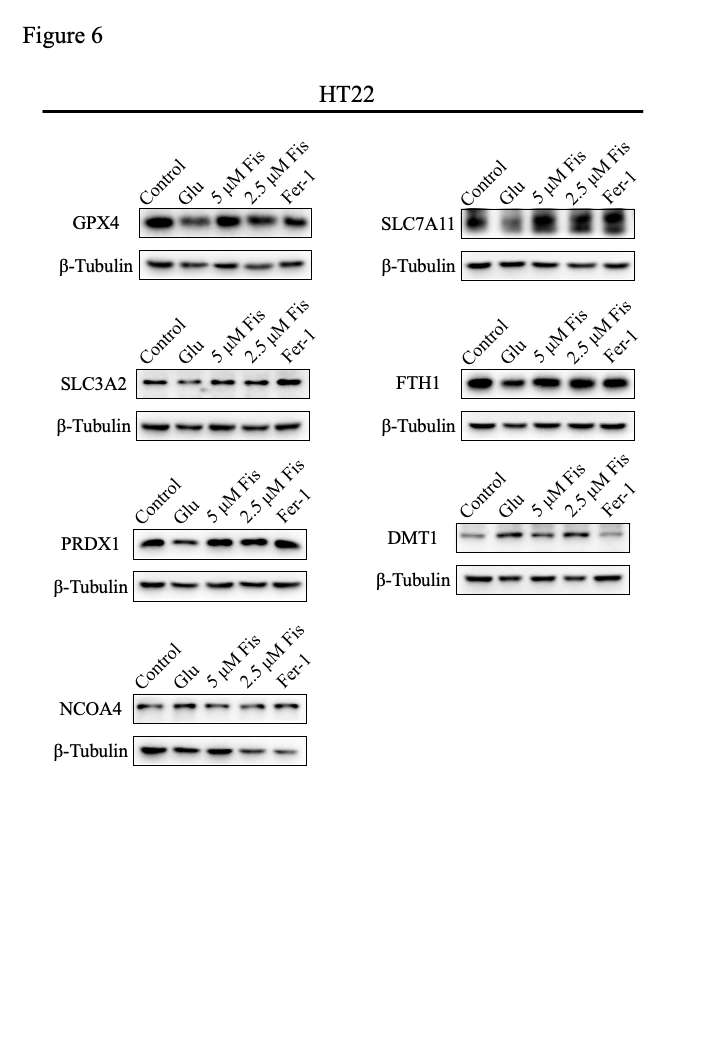


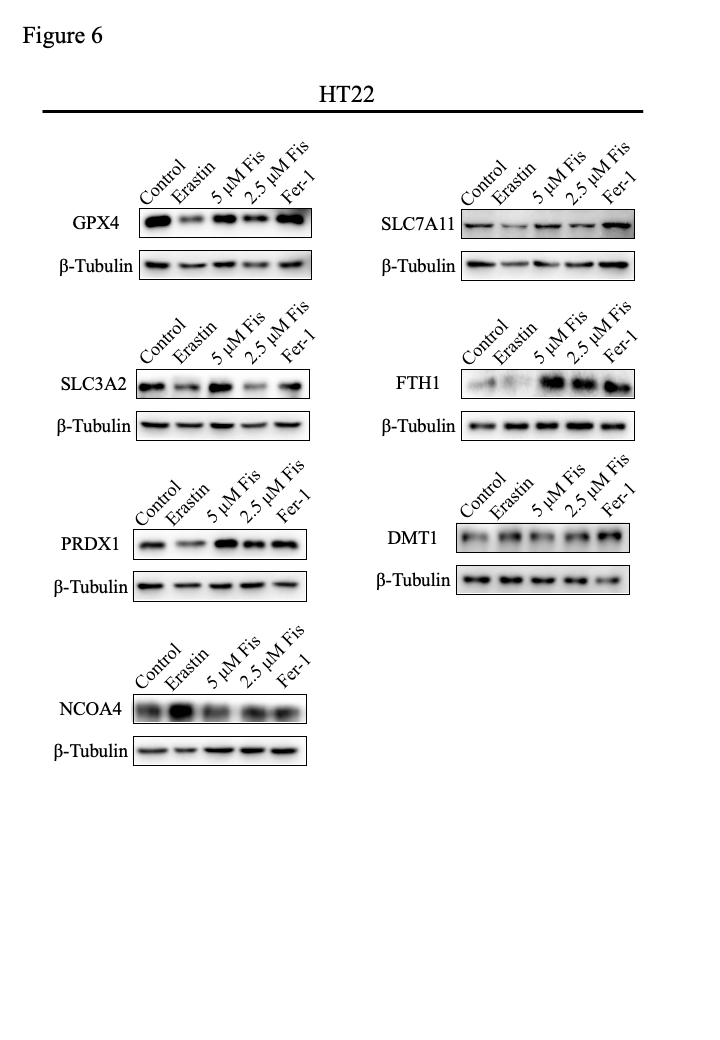


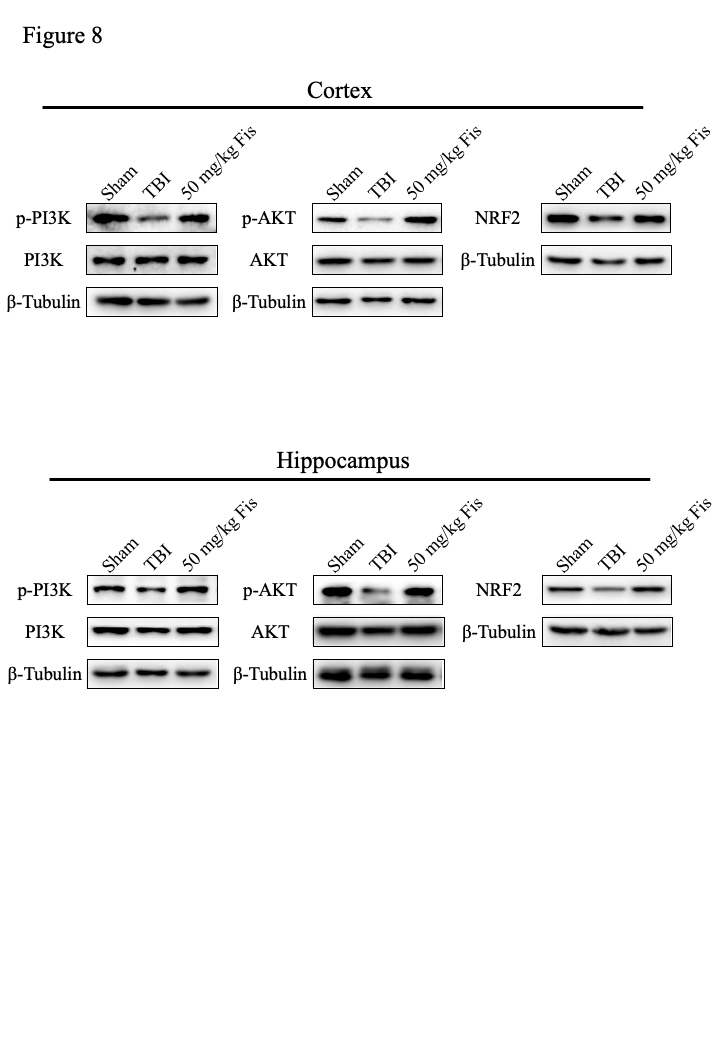

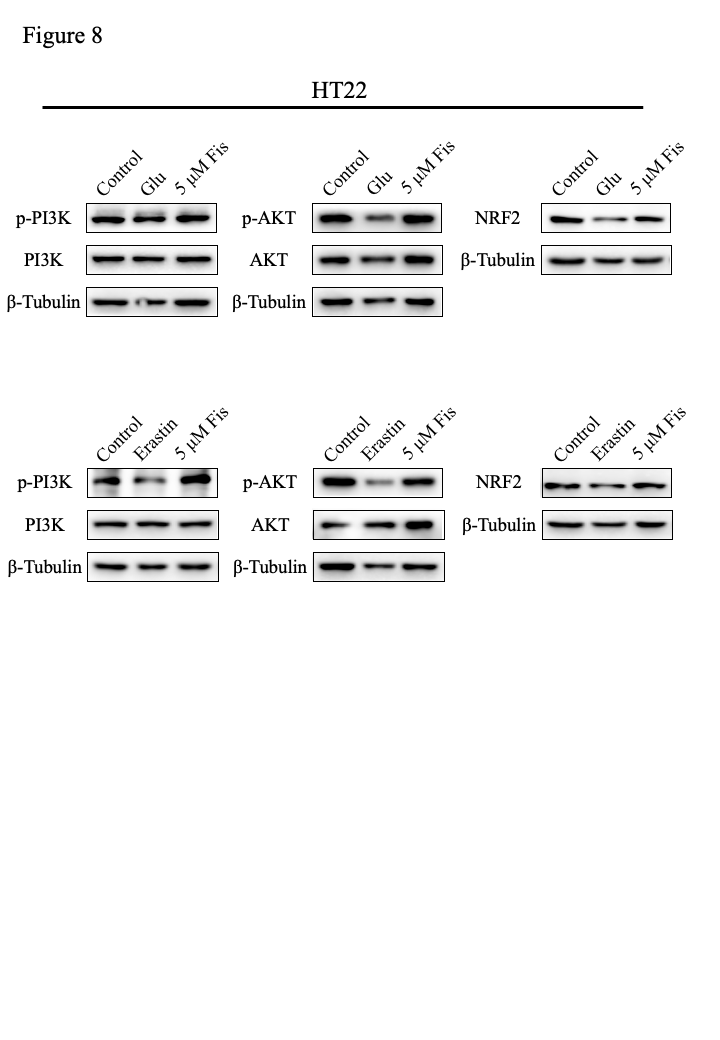


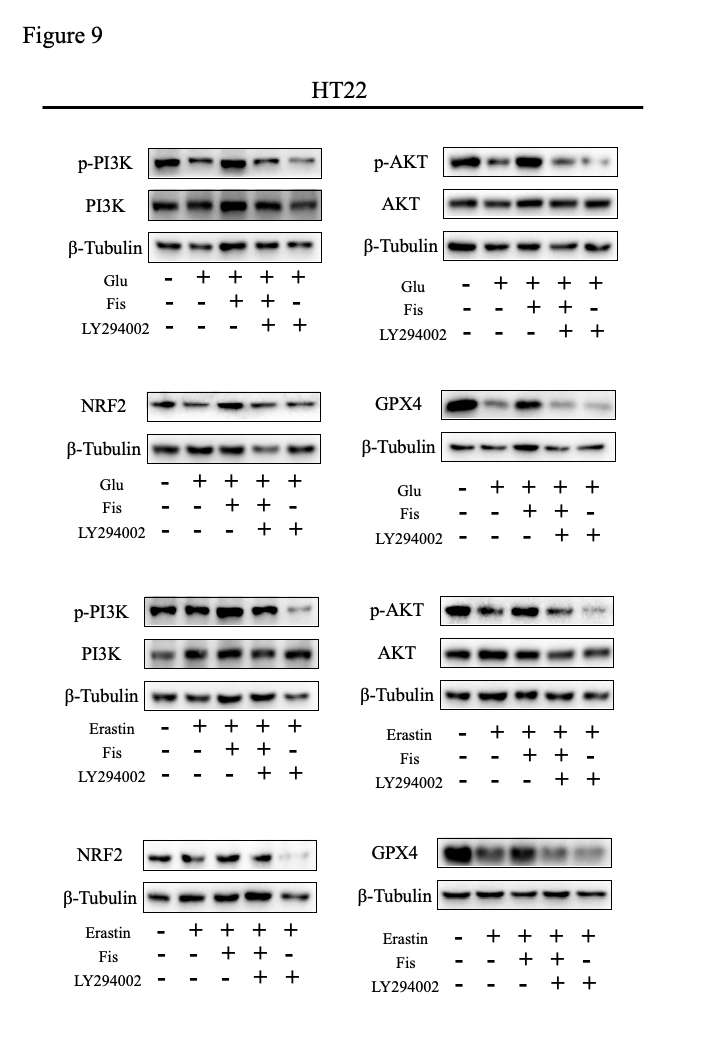


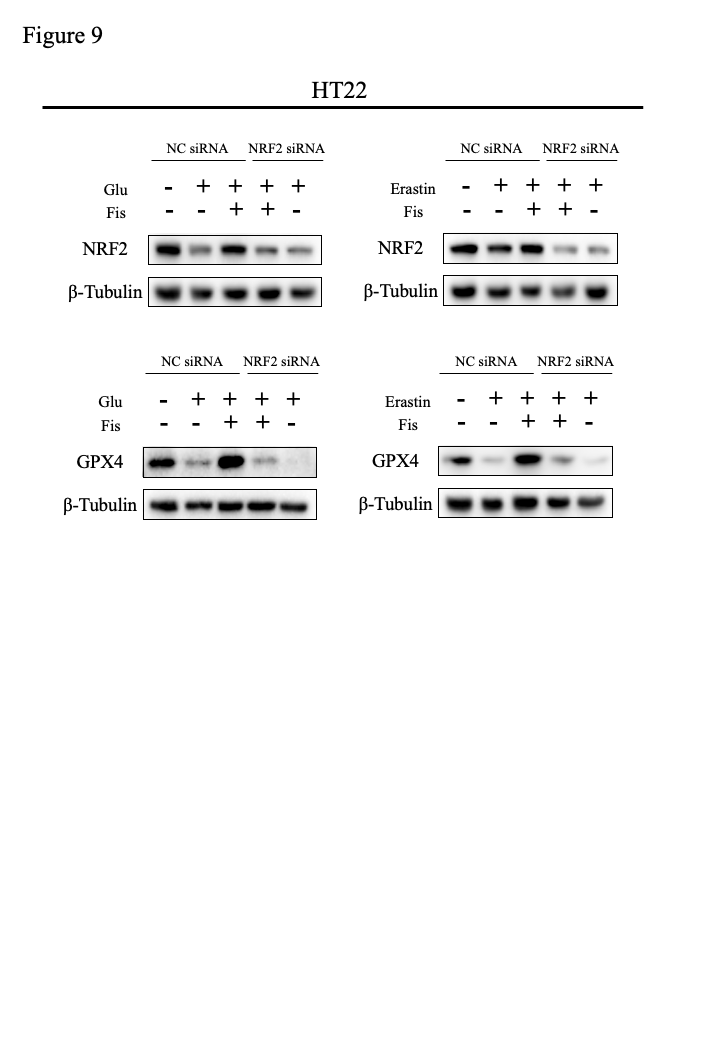

Supplement: Supplementary file 1 [file DataSheet1.docx]
